# Gender Differences in Diabetic Kidney Disease: Focus on Hormonal, Genetic and Clinical Factors

**DOI:** 10.3390/ijms22115808

**Published:** 2021-05-28

**Authors:** Annalisa Giandalia, Alfio Edoardo Giuffrida, Guido Gembillo, Domenico Cucinotta, Giovanni Squadrito, Domenico Santoro, Giuseppina T. Russo

**Affiliations:** 1 Department of Clinical and Experimental Medicine, University of Messina, 98125 Messina, Italy; agiandalia@yahoo.it (A.G.); domenico.cucinotta@unime.it (D.C.); giovanni.squadrito@unime.it (G.S.); 2 Unit of Nephrology and Dialysis, Department of Clinical and Experimental Medicine, University of Messina, 98125 Messina, Italy; alfiogiuffrida91@libero.it (A.E.G.); guidogembillo@live.it (G.G.); domenico.santoro@unime.it (D.S.); 3 Department of Biomedical, Dental, Morphological and Functional Imaging Sciences, University of Messina, 98125 Messina, Italy

**Keywords:** gender, sex, diabetic kidney disease, estrogens, gene polymorphisms

## Abstract

Diabetic kidney disease (DKD) is one of the most serious complications of both type 1 (T1DM) and type 2 diabetes mellitus (T2DM). Current guidelines recommend a personalized approach in order to reduce the burden of DM and its complications. Recognizing sex and gender- differences in medicine is considered one of the first steps toward personalized medicine, but the gender issue in DM has been scarcely explored so far. Gender differences have been reported in the incidence and the prevalence of DKD, in its phenotypes and clinical manifestations, as well as in several risk factors, with a different impact in the two genders. Hormonal factors, especially estrogen loss, play a significant role in explaining these differences. Additionally, the impact of sex chromosomes as well as the influence of gene–sex interactions with several susceptibility genes for DKD have been investigated. In spite of the increasing evidence that sex and gender should be included in the evaluation of DKD, several open issues remain uncovered, including the potentially different effects of newly recommended drugs, such as SGLT2i and GLP1Ras. This narrative review explored current evidence on sex/gender differences in DKD, taking into account hormonal, genetic and clinical factors.

## 1. Introduction

Diabetic kidney disease (DKD) is one of the most common microvascular complication of diabetes mellitus (DM), affecting ~30% of subjects with type 1 (T1DM) and ~40% of those with type 2 (T2DM) [1].

DKD is diagnosed according to the presence of albuminuria, reduced estimated glomerular filtration rate (eGFR), or both [2].

In the last few decades, research on DKD has witnessed enormous activity, a revolution encompassing epidemiology, diagnosis, clinical manifestations, risk factors and treatment options [3]. It has become clear that while in T1DM individuals, the natural history of DKD progresses from microalbuminuria, the first sign of renal impairment, to macroalbuminuria, and eventually to the decline of GFR toward end stage renal disease (ESRD), the path in T2DM is more heterogeneous. Thus, T2DM patients may present with impaired eGFR even only a few years after diagnosis, and they may progress to ESRD without ever developing albuminuria [4]. Accordingly, the recent advances in DKD’s pathophysiological and clinical aspects have prompted the use of the term “DKD” to include all types of renal injury occurring in diabetic individuals: the classical albuminuric phenotype, the “nonalbuminuric renal impairment” and the “progressive renal decline” [5]. These advances have been highlighted in a joint document of the Italian Diabetes Society (SID) and the Italian Society of Nephrology (SIN), providing an extensive review of the available evidence as well as updated treatment recommendations [5].

However, whether these phenotypes and their evolution and/or management have the same course in DM men and women is still a matter of debate.

Considering sex- and gender-specific aspects is one of the first and simpler step toward personalized and patient-centered care also in the management of DM and its complications. Gender medicine analyzes the differences between men and women in human physiology, pathophysiology, and the clinical features of diseases, specifically evaluating the impact of sex as a biological and functional marker, and that of gender, which refers to a complex interrelation and integration of sex with psychological, social, ethnical and cultural behavior. Despite over 20 years of gender medicine and the insistent recommendations of scientific societies and research institutions [6], sex-based differences in biology, genetics, biomedical and clinical aspects of major diseases, including DM and its complications, are still poorly explored so far, a gap that may impair the efficacy of our diagnostic and therapeutic efforts, ultimately exposing patients to unwanted outcomes [7].

In this review, we explored potential sex- and gender-based differences in DKD prevalence and evolution, risk factors, clinical manifestations and treatment options, including the role of hormonal and genetic factors.

## 2. Gender Differences in the Prevalence of DKD and its Phenotypes

Gender-related differences have been reported in non-diabetic chronic kidney disease (CKD) [8]. Overall, CKD seems to have a higher prevalence in women than in men [9,10,11]. However, a review including a large number of studies found 38 studies reporting a higher CKD prevalence in women and 13 among men [12]. On the contrary, the risk of progression to ESRD appears to be higher among males [13,14]. A recent analysis from the nationwide Swedish Renal Registry-CKD (SRR-CKD), showed that among adult patients with incident CKD stage G3b-5, women had a lower risk of CKD progression (sub hazard ratio [SHR] 0.88 (0.85–0.92)), and a lower all-cause (SHR 0.90, 95% CI 0.85–0.94) and cardiovascular mortality (SHR 0.83, 95% CI 0.76–0.90), compared to men [15]. Accordingly, a large meta-analysis confirmed a more rapid decline in men than in women with non-diabetic CKD [16]. Women live longer than men and age and post-menopausal status appear to modify the association between sex and nondiabetic kidney disease [17]; in this regard, Jafar et al. reported that old post-menopausal women had a faster renal progression compared to age-matched men [18].

How CKD is defined may also play a relevant role in determining the effect of gender on CKD incidence and progression risk. Thus, when using eGFR-based definitions of CKD, the incidence of CKD was significantly higher in women than men [19].

In cohorts of DM subjects, several large epidemiological studies have explored sex differences in the prevalence of DKD and its phenotypes, specifically evaluating low eGFR, micro- or macroalbuminuria or both (Table 1). Studies performing a separate gender analysis varied in design, sample size, type of diabetes, length of follow-up, and how DKD was reported, as shown in Table 1 [13,20,21,22,23,24,25,26,27,28,29,30,31,32,33,34,35,36,37,38,39,40,41,42,43,44,45,46,47,48,49,50,51,52,53,54,55,56,57,58] and in two recently published reviews [59,60].

A recent meta-analysis of 10 studies including data from >5 million subjects evaluated the relative effect of diabetes on CKD and ESRD in women compared with men. The pooled adjusted risk ratio of DKD was 3.34 (95 % CI 2.27, 4.93) in women and 2.84 (95 % CI 1.73, 4.68) in men, without any difference in diabetes-related risk of DKD, with the exception of ESRD [61]. Furthermore, in the SURDIAGENE study, survival without ESRD was higher in T2DM women than in men, who showed an higher risk of steeper eGFR decline [31].

Among the studies that evaluated DKD phenotypes specifically in T2DM cohorts, the UK Prospective Diabetes Study 74 (UKPDS) found that T2DM men had a higher risk of microalbuminuria, while T2DM women were at higher risk of developing low eGFR (<60 mL/min/1.73 m^2^) [24]. Similarly, a population study from UK showed a higher prevalence of impaired eGFR in DM women compared to men [62], and a higher prevalence of the non-albuminuric phenotype among T2DM women was also reported in the large cohort from the RIACE study, in Italy [63]

Age may impact the associations among T2DM, gender and DKD risk. In T2DM patients, the prevalence of both eGFR and albuminuria increases with age, with male sex also positively associated with albuminuria rise and negatively with eGFR impairment in older subjects [64].

The impact of age (<60 and ≥60 years) and gender on the prevalence of DKD, advanced DKD (eGFR <30 mL/min/1.73 m^2^), and common risk factors, was specifically analyzed in the 4839 participants of the Pathways Study [26]. This study showed that women had, overall, a 28% decreased OR of DKD (OR 0.72, 95% CI 0.62–0.83), but a higher prevalence of advanced DKD (OR 1.67, 95% CI 1.05–2.64). Although the prevalence of microalbuminuria was higher among men with T2DM, women presented a greater risk of advanced renal dysfunction and higher prevalence of common DKD risk factors, with these differences being most evident amongst older subjects.

Male gender also appears to be an independent risk factor for DKD incidence, especially when the albuminuric phenotype is taken into account. A prospective observational study of 191 T2DM patients followed for a median period of 5.8 years [20] found that male sex was the second risk factor after albuminuria for the development of incipient or overt DKD. Moreover, an association between sex and incident DKD was also found in 1464 patients with diabetes and normal renal function at baseline, followed-up for almost 10 years [65].

**Figure 1:**
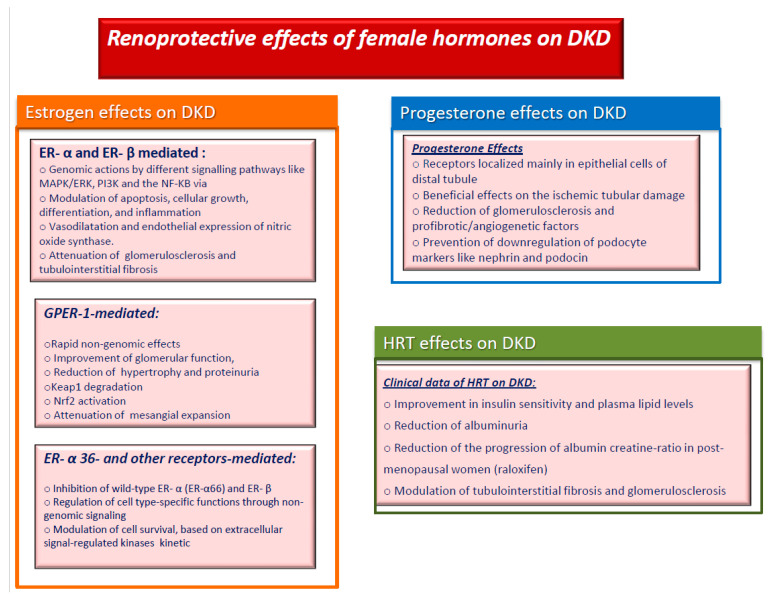
Renoprotective effects of female hormones on diabetic kidney disease. Abbreviations: DKD, diabetic kidney disease; ER, estrogen receptor; HRT, hormone replacement therapy; GPER-1, G protein-coupled estrogen receptor 1.

Risk factors for prevalence, incidence and progression of DKD and its phenotypes have been recently evaluated in the large cohort of the AMD Annals Initiative, an observational study including >400,000 T2DM and >25,000 adult T1DM subjects, from 251 diabetologists’ centers collected across Italy (Table 2). Figure 1 [66,67,68,69,70,71,72,73] shows gender-specific DKD phenotypes distribution in T1DM and T2DM from the AMD Annals Initiative. DKD, defined as eGFR <60 mL/min and/or micro- macroalbuminuria, affected 47.3% T2DM men (29.8% isolated micro- macroalbuminuria, 6.6% isolated low eGFR, 11% both) and 43.8% T2DM women (18.3% micro-macroalbuminuria, 14.5% isolated low eGFR, 11.2% both) [66]. Furthermore, when predictors of DKD were analyzed in >120,000 T2DM patients over a 4 year follow-up period, male gender was a positive risk factor for the incidence of albuminuria and a negative risk factor for the development of eGFR decline (eGFR <60 mL/min) [72].

Although the available information is still not conclusive, the overall epidemiological data indicate that the risk of developing DKD is higher in DM men, who also have a higher risk of DKD progression. However, when DKD specific phenotypes are taken into account, DM men are at higher risk of developing the albuminuric phenotype, while women are at higher risk of eGFR impairment, and of developing ESRD, especially at older ages. These differences seem to apply to both type of diabetes, with important implications for the diagnosis and management of DKD in the clinical practice.

The reasons behind these reported sex and gender-disparities in DKD are still largely unknown, but hormonal or genetic differences, as well as differences in the prevalence or impact of major risk factors seem to play a relevant role.

## 3. Impact of Female Sex Hormones on DKD

Sex hormones play an important role in the pathophysiology of diabetes and its complications, especially in DM women, who seem to lose the protective effects of estrogens on the cardiovascular bed, even before menopause. In the last few decades, the pleiotropic effects of estrogens beyond those on the reproductive system have been objects of intense research [74,75,76], including their potential role in DKD (Figure 1). The activity of estrogens is closely related to the presence of specific receptors that are ubiquitously localized, with particular reference to the vascular district and endothelial cells [77]. Estrogens and their metabolites operate through a classical pathway with nuclear Estrogen Receptors (ERs) such as ER-α and ER-β [78,79] exerting their genomic actions. These effects are guaranteed by different signaling pathways such as MAPK/ERK, PI3K and the important NF-KB via [80]. These pathways lead to decreased apoptosis processes, cellular growth, differentiation, and inflammation [81,82]. The ER-α and ER-β are localized in several areas of crucial importance. One of these is the hypothalamus, especially the nuclei arcuate, paraventricular, lateral, and ventral regions, where estrogens produce relevant effects on food intake and thirsty [83] Another site is the skeletal muscle, where both ER-α and ER-β are expressed and contribute to glucose homeostasis, reducing the expression of GLUT4 [84]. This role has also been confirmed in studies on mice with ER-α knockout; these mice showed insulin resistance and alteration in glucose levels [85].

Several receptors have been identified in adipose tissue, whereas only ER-α is expressed in brown adipose tissue [86], suggesting its key-role in obesity onset [87]. ER-α is also the most predominant estrogenic receptor in the liver [88] while during the fetal phase ER-β is more represented [89]. The former has especially predominant anti-inflammatory effects on the liver [90]. The cardiovascular system is not exempt from ERs’ presence, and their interaction with estrogens can ameliorate heart failure and inhibit apoptosis and fibrosis [91]. Furthermore, activation of ER-β pathway leads to the reduction of cardiac fibrosis in women [92].

Experimental studies demonstrated that ER-α expression is abnormally represented in the diabetic kidney [93]. Estrogen’s most important metabolite is the 17 β Estradiol (E2) [94]; in healthy conditions, this metabolite acts as a vasodilator, increasing the endothelial expression of nitric oxide synthase and resulting in phosphorylation and nitric oxide production via the ER-α receptor [95]. Moreover, E2 seems to attenuate glomerulosclerosis and tubulointerstitial fibrosis [96]. In animal models estrogens seem to counter fibrosis and apoptosis in the kidney [97], while testosterone promotes pro-inflammatory, pro-apoptotic and pro-fibrotic processes [98,99,100]. These findings are partially in contrast with evidence from human studies showing an association of oral contraceptives and estrogen replacement therapy with an increased risk of microalbuminuria and kidney function decline [101,102].

Estrogens may exert their effects even through another receptor, associated with protein G, the G protein-coupled estrogen receptor 1 (GPER-1), which explains their rapid non-genomic effects [103]. This is a membrane receptor encoded by the GPER gene located at chromosome 7p22.3, and its expression has been demonstrated in the hypothalamus, hypophysis, adrenal gland, ovary and particularly in the renal pelvis [104,105]. Some studies have also evidenced a predominant expression of GPER-1 in renal tubular cells [106,107], and therapy with GPER-1 agonist in female mice with salt-sensitive disease has been reported to improve glomerular function and hypertrophy and to reduce proteinuria [108], thus suggesting a key role of this receptor in kidney disorders and, probably, in DKD. This hypothesis is further supported by data coming from the sustained use of icarine, a GPER-1 activator. This metabolite seems to improve the nephropathy of T1DM mice, through GPER mediated p62-dependent Keap1 degradation and Nrf2 activation, also attenuating mesangial expansion [109]. GPER is implicated in different pathways by several receptors such as serotonin 1A receptor [110,111,112,113]. It also works with GPER/TGF-β1 via inducing cellular proliferation or increasing the expression of type IV collagen [114] and NO production [115].

Other membrane receptors were identified: ER-α 36, a variant of ER-α and ER-β. ER-α 36 is another splicing variant; it plays an important role, inhibiting wild-type ER-α (ER-α66) and ER-β, being involved in the resistance of breast cancer to hormonal treatment [116] and in testosterone-initiated carcinogenesis [117].

The experimental data on estrogen supplementations in murine models of diabetes also seem to confirm the role of female hormones in renal protection.

Thus, Wells et al. demonstrated a reduction in circulating estrogen levels and an increase in the renal ER-α—ER-β expression ratio in diabetic rats. Supplementation with E2 restored this rate, supporting the hypothesis of nephroprotection exerted by estrogens in DM [118]. Furthermore, in diabetic mice, estrogen pellet implantation was able to inhibit glomerulosclerosis, collagen IV deposition and albuminuria, even in animals with an advanced stage of renal injury, suggesting the efficacy of E2 treatment in the reduction of DKD progression [119]. On the other hand, the inhibition of estradiol synthesis using the anastrozole, an aromatase inhibitor, partially attenuated renal injury in male streptozotocin-induced diabetic rats [120].

Both the levels of E2 and the androgen-to-E2 ratio seem to be crucial factors for progression of renal injury in diabetic subjects; estrogenic activity is modulated by androgens, probably through the accessibility of ER to E2. In humans, DKD is associated with an increase in estrogen concentration and a decrease in testosterone levels in male patients [121], but not in women [122,123].

The potential beneficial effect of hormonal replacement therapy (HRT) on DKD has also been explored, with some studies supporting the efficacy of estrogenic therapy in improving insulin sensitivity, plasma lipid levels and creatinine clearance in postmenopausal diabetic women [124,125,126]. Maric et al. reviewed the current literature in this field, underlining the protective role of estrogens in DKD and the role of oral supplementation of 17 β estradiol in attenuating its evolution [127].

Further investigations have shown that treatment with 17 β estradiol decreased albuminuria, tubulointerstitial fibrosis and glomerulosclerosis in the diabetic population [128]. Oral therapy with selective estrogen receptor modulators showed similar benefits.

Additionally, a provisional regimen with raloxifene, a selective estrogen receptor modulator, reduced albuminuria levels in post-menopausal women with T2DM [129]. Furthermore, improvements of important kidney outcomes, including a reduction in the progression of the albumin–creatinine ratio in post-menopausal T2DM women [130] has been reported in a randomized trial with raloxifen.

Accordingly, the oral combination therapy with estradiol and norgestrel improved eGFR and proteinuria in T2DM postmenopausal women with hypertension, and this nephroprotective action was not related to the modification of conventional risk factors such as blood pressure and lipids [126]. Conversely, a recent meta-analysis demonstrated the short-term benefits of HRT on lipid profile in young women with CKD, although a reduction in CV morbidity and/or mortality was not observed [131].

Vitamin D could also exert a synergetic action with estrogens in renal protection and diabetes control [132]. Thus, vitamin D acts as a real steroid hormone and its level is influenced by estrogen status [133]. It exerts a modulating action at both tubular [134] and glomerular level [135], with peculiar protective action in DKD [136]. Moreover, pre- and post-menopausal DM women with an adequate vitamin D status seem to have a better glycemic control [137]. Estrogens interfere with vitamin D immunomodulatory activities [138] and, in turn, vitamin D down-regulates aromatase action, with a reduction of the adverse events linked to peripheral estrogen overexpression [139]. Combined therapy with vitamin D supplementations and sex steroids seems to protect endothelium integrity, contrasting the cardiovascular damage that contributes to CKD and DKD progression [140,141].

Multiple mechanisms behind the reno-protective effects of oral estrogen supplementation in DM women have been reported. Chronic hyperglycemia leads to an increase of reactive oxygen species (ROS), and an impairment of nitric oxide (NO) secretion [142]. Additionally, the polyol pathways are involved in the DKD genesis [143] Another pathway of glycemic damage is represented by the accumulation of Advanced Glycation End Products (AGEs), which are derived from nonenzymatic glycosylation of product proteins or lipids [144,145]. AGEs reduce the efficiency of anti-oxidant systems, downregulating several protective molecules, such as AGER1 and SIRT1 [146,147].

E2 therapy can interfere with these pathways at different levels. It reduces the expression of transforming growth factor-beta (TGF β), AT1 receptors, and endothelines with a decreased production of collagen (especially I and IV) [148,149,150,151] and a reduction of apoptotic phenomena [152]. The increased activity of nitric oxide synthase at the glomerular level may be another effect of estrogens in the kidney, improving vascular permeability and glomerular function [153].

A study on ovariectomized rats has shown an increased expression in glomerular tissue of SIRT1 mRNA in those treated with E2. In addition, E2 increased ER α -mRNA expression in the glomerular mesangium and reduced the fibrotic process and TGF-β levels [154]. TGF-β plays a key role in the genesis of DKD, increasing the production of extracellular matrix, and that of collagen IV in the podocytes and expanding the mesangial area [155,156]. Its expression is upregulated by a state of chronic hyperglycemia, leading to glomerulosclerosis [157,158,159], and TGF-β levels are usually increased in men and reduced in women [160], thus indirectly suggesting a crucial role of estrogens in regulating TGF-β. Accordingly, ER-α, the major estrogenic receptor expressed at the renal level, has also been shown to bind to several target molecules [161].

Other metabolic pathways are involved in the regulation of TGF β levels, such as the protein kinase system (CK2) [162] and the renin–angiontensin–aldosterone system (RAAS) that increase TGF-β production [163], and estrogens have been also reported to reduce the activity of RAAS and, therefore, stimulate TGF-β [164].

Beyond the role of estrogens, progesterone also seems to play an important role in kidney protection. Thus, progesterone receptors are mainly localized in the epithelial cells of distal tubule [164], both in the medulla and cortex kidney of male and female subjects [165]. Loss of renal function related to the ageing process and damage to of the proximal tubule could be prevented by the administration of estrogen alone or even by the addition of progesterone replacement therapy [166], which also demonstrated beneficial effects on the ischemic tubular damage [167], further supporting their role in nephroprotection.

Accordingly, Baha et al. highlighted that the administration of progesterone for 10 weeks in ovariectomized diabetic mice improves the different outcomes of diabetic nephropathy, reducing glomerulosclerosis and profibrotic/angiogenetic factors (TGF-β, vascular endothelial growth factor -A, type 1 receptor of angiotensin II), downregulating podocyte markers such as nephrin and podocin [168].

In spite of this increasing experimental evidence of the renoprotective role of estrogens alone or in combination with progesterone, large prospective trials with HT in postmenopausal T1DM and T2DM women with variable stages of CKD are still needed in order to evaluate their possible role in the treatment of this high-risk population in future.

## 4. Impact of Sex Genes Interactions on DKD

Sex may impact the effect of genes at different levels. Recent evidence supports the role of sex chromosomes on renal impairment in non-diabetic individuals, as reported for Alport syndrome, arising from a mutation in *COL4A5* on chromosome X [169], and showing a different renal prognosis in affected males and females [170,171].

However, the influence of sex is far more complex, and sex is an important modifier of the influence of genetic background on several chronic diseases, including CKD.

Overall, the genetic heritability of CKD has been estimated to range from 30 to 75% [172,173] and several lines of evidence confirmed the relevant role of genetic predisposition in the initiation and progression of renal complications both in T1DM and T2DM subjects. Epidemiological studies have revealed familial clustering of DKD in both types of diabetes [174,175], and a relevant influence of ethnical background [176,177,178]. More than 150 genes have been associated with DKD in T1DM and T2DM, although with a different biological relevance, and most of them have been identified through genomewide association studies (GWAS) [179,180,181,182,183].

A recent GWAS analysis on DKD, involving large T2DM and T1DM cohorts and taking into account eight complementary dichotomous and quantitative DKD phenotypes, identified a novel loci (near GABRR1, rs9942471) specifically associated with microalbuminuria in European T2DM case subjects only, with no signal in Asian diabetic subjects or in those with T1DM irrespective of ethnical origin [184]. These findings indicate that a phenotype-, ethnicity- and type of diabetes-driven analysis may be more specific in the identification of genetic susceptibility to DKD. Accordingly, in T1DM, a GWAS analysis of 19,406 subjects using various definitions of DKD, based on renal function and albuminuria, recognized 16 loci, including protective variants (the rs55703767 minor allele, Asp326Tyr) and a variant (rs55703767), on the collagen type IV alpha 3 chain (*COL4A3)* gene, with the most significant association with DKD in T1DM patients [185].

Furthermore, the minor C allele of rs17389016 of the 11β-Hydroxysteroid dehydrogenase 1 (HSD11B1) was recently associated with the “fast decliner” phenotype and overt DKD in a cohort of 466 T1DM subjects (OR = 2.10; CI 95% = 1.14–3.89; *p* = 0.018) [186].

Despite this overwhelming evidence, the identified genes and single nucleotide polymorphisms (SNPs) only explain a minor part of the genetic susceptibility to DKD. Thus, epigenetic mechanisms, i.e., DNA methylation, chromosome histone modification and noncoding RNA (ncRNA) regulation [187,188] and their potential interactions with personal or environmental factors, including sex, may also play an important role [189].

Although several genetic loci in genes implied in the RAAS, inflammation, oxidation, glucose and lipid metabolism have been associated with DKD, the potential role of sex–gene interaction has been evaluated for only a few of them [8].

In one of the first studies of gene–gender interaction on DKD, a case–control study from the Joslin Diabetes center, the M235T variant in the angiotensinogen gene, which is associated with a greater expression of this gene, increased DKD risk only in T1DM men [190].

In T2DM study subjects from the Health Professionals Follow-Up Study (HPFS) and the Nurses’ Health Study (NHS), sex-specific associations were found between the angiotensin II type 1 receptor gene AGT1R 1166 C-allele and AGT 235T and coronary heart disease (CHD), whereas the AGT1R T573 C-allele variant was not associated with CHD or DKD [191]. Furthermore, a case–control study in a large cohort of T1DM patients from Denmark, Finland, France and Sweden, found that the AA genotype of the rs5186 AGTR1 polymorphism significantly increased the DKD risk in male patients (OR = 1.27; 95% CI = 1.02–1.58, *p* = 0.03), after adjustment for multiple confounders, whereas no significant associations were noted in women [192].

Sex differences were also noted for common variants in carnosinase genes on chromosome 18q, CNDP1 and CNDP2 [193], with the 5-leucine repeat (5L-5L) variant of the CNDP1 gene being associated with a reduced prevalence of DKD in T2DM women [194], whereas the rs12604675-A variant in CNDP1 was shown to confer higher susceptibility to overt proteinuria in T2DM women from Japan [195].

The common angiotensin-converting enzyme (ACE) polymorphism (I = insertion, D = deletion) has also been extensively studied as a DKD susceptibility gene in both T1DM and T2DM. While this variant was not able to explain the observed gender differences in ESDR occurrence in black DM individuals [196], it seemed to have an independent impact on survival in DM patients on dialysis [197]. Furthermore, T2DM women carriers of the ACE D allele were found to be at increased risk of DKD progression, whereas no difference was found in T2DM men, even after adjustment for multiple confounders [198].

Sex–genes interactions were also reported for genes implied in inflammation and oxidation, as well as in other DKD-related risk factors.

Significant interaction with sex was also noted for the *IL-6 (rs1800795)* genetic variant in a study on predictive genetic models of microalbuminuria in relatives of subjects with DKD [199].

In 1120 T1DM subjects (529 men and 591 women), the *SNP rs11915160 SOX2* gene, located in chromosome 3q26.33, was significantly associated with DKD and ESRD in women but not in men, with a combined effect with the adiponectin promoter polymorphism rs266729 [200]. Sex-specific associations were also reported for two SNPs in the regulatory regions of CYBB (NOX2, coding, respectively, for superoxide-generating nicotinamide adenine dinucleotide phosphate-oxidase 2) and GPX4 (glutathione peroxidase 4), involved in the redox status. The minor *A-allele of CYBB rs6610650* was associated with DKD in women, whereas the *minor T-allele of GPX4 rs713041* showed an inverse association with DKD in T1DM men of South America and European origins [201].

T1DM male carriers of the *59029G allele* and those with the *32-bp* deletion on the *secreted (RANTES) receptor gene (CCR5)* variant, which is associated with the diminished expression of CCR5 on immunocompetent cells, had a greater risk of DKD, compared with non-carriers. Furthermore, the distribution of the combined haplotypes with these two variants differed significantly in men with and without DKD, but not in women [202].

GWAS studies in T1M cohorts also identified sex specific associations with DKD susceptibility variants. Thus, a GWAS study in the large cohort of the Finnish Diabetic Nephropathy (FinnDiane) Study found that a common variant, the rs4972593 on chromosome 2q31.1, that was associated with ESRD in women but not in men, and this was confirmed in a meta-analysis of three independent T1DM cohorts [203]. Notably, this variant (rs4972593) is able to interact with ERα, modulating the expression of genes implicated in glomerular function and cell proliferation, thus potentially contributing to the sex-specific protection against ESRD [203].

Another study conducted on three cohorts including T1DM of European descent found that the IGF2BP2 polymorphism, a variant protein that binds to 5’-UTR of the imprinting IGF2 gene, was associated with DKD only in male T1DM subjects. In the same cohorts, a genetic interaction between IGF2BP2 and IGF2 gene was also identified, suggesting a protective role against DKD in male T1DM subjects [204].

In T2DM women, the *PNPLA3 rs738409* polymorphism, a genetic risk factor for non-alcoholic fatty liver disease, was associated with impaired eGFR values and DKD prevalence, irrespective of the presence of NAFLD and common cardio–renal risk factors [205].

Polymorphisms in cholesteryl ester transfer protein (CETP) gene, a key enzyme in triglyceride metabolism, were also investigated as genetic risk factors for DKD. In a group of T2DM women, followed up for ~9 years, the Taq1B variant was not associated with the risk of developing DKD, whereas it predicted the onset of diabetic retinopathy [206].

Conversely, another study, conducted on a total of 3023 Taiwanese individuals (1383 without and 1640 with T2DM) found that the A-allele of rs1800775 in the CETP gene was significantly related to a lower DKD risk (OR, 0.78; 95% CI, 0.64‒0.96) [207]. Unfortunately, no gene–gender specific analysis was conducted in this study and ethnic differences may also have played a role in differentiating these results.

No sex differences were found according to five SNPs in other genes involved in lipid metabolism such as the SLC2A9 and ABCG2 genes in DKD [208].

Genetic background has a relevant role in increasing or decreasing the risk of developing DKD; however, the relative importance of each genetic variant seems to vary according to type of diabetes, study design, DKD phenotypes (albuminuric/low eGFR), and ethnic background. Overall, the available evidence suggests that sex should be considered among the variables potentially influencing the impact of genes on DKD risk. Thus, when a sex-specific analysis was conducted, some of these genetic risk factors were exclusively or more strongly associated with DKD in either DM men or women (Table 3) [180,181,182,183,184,185,186,189,190,191,192,193,194,195,198,200,201,202,203,204,205,207]. Future research should persevere to confirm sex-specific associations and basic research should investigate the mechanisms behind these observed results.

## 5. Gender Differences in DKD Risk Factors and Renoprotective Drugs

Cardiovascular disease (CVD) and DKD share several common risk factors, including hyperglycemia, high blood pressure (BP) values, atherogenic dyslipidemia, obesity, inflammatory markers, high uric acid levels and others. Furthermore, both albuminuria and low eGFR values independently increase CVD morbidity and mortality risk in diabetic individuals [209].

Sex and gender differences have been largely reported in CVD and its major risk factors, both in T2DM and T1DM [70,210,211,212,213,214,215,216,217,218]. Thus, while the CVD risk is overall higher in DM men than in women, the relative risk of developing CVD complications is 2-4 times higher in DM females than in males [210,211,212]. Ageing seems to reduce the impact of diabetes on CVD risk, being particularly high in subjects 35-59 years [213,214].

Recent data from the UK estimated the excess CVD risk related to T2DM being approximately 50% higher in women (HR 1.96 (95% CI 1.60, 2.41)) than in men (HR 1.33 (95% CI 1.18, 1.51)) [215], although some more contemporary data attenuated these findings [216].

Notably, an Italian cohort of >11,000 T2DM subjects, followed up for 4 years, reported that, the impact of common risk factors on coronary heart disease (CHD) was different in T2DM men and women, with microvascular complications, including DKD, being a stronger risk factor in females [217].

Thus, sex and gender differences were also reported for several DKD risk factors, with T2DM women generally showing a greater prevalence of out-of-target values [70,218].

In the AMD Annali Initiative, women were 14% more likely than men to have HbA1c >9.0, 42% more likely to have LDL-cholesterol ≥130 mg/dl, and 50% more likely to have BMI ≥30 kg/m^2^ despite appropriate treatment [1]. Furthermore, older T2DM women were those at the highest risk of uncontrolled lipid values [219].

Beside LDL-C levels, also atherogenic dyslipidemia, i.e., high triglycerides (TG) and low HDL-C values, the typical lipid profile in subjects with insulin-resistance and T2DM, has been recognized as an independent risk factor for DKD [220]. In 15,362 Italian T2DM patients, with eGFR ≥60 mL/min/1.73 m^2^, normoalbuminuria, and LDL-C ≤130 mg/dL at baseline, followed-up for 4 years, low HDL-C and high TG levels were independent risk factors for the development of DKD, defined as either low eGFR (<60 mL/min/1.73 m^2^), eGFR reduction >30% and/or albuminuria. After stratification for multiple risk factors, the association of low HDL-C levels with low eGFR was more pronounced for male gender, whereas no other sex interactions were noted for high triglycerides and low eGFR risk and for both lipid fractions and microalbuminuria (MAU) [221]. Notably, another Japanese observational cohort study found an association of atherogenic dyslipidemia with a higher risk of developing DKD in men only [222]. The heterogeneity of HDL particles in their lipid and protein composition, as well as in their function may have contributed to these results. In order to clarify this issue, we evaluated the effect of atherogenic dyslipidemia, the HDL subclasses distribution and the common cholesteryl ester transfer protein (CETP)TaqIB variant on the incidence or the progression of DKD and diabetic retinopathy (DR) in a group of T2DM women followed up for ~9 years. In this study, atheroprotective HDL subclasses together with BMI and LDL/HDL ratio were associated with an increased risk of developing DKD; although these associations were attenuated at multivariate analysis [206]. BMI, LDL/HDL ratio and low levels of α-1 HDL particles were associated to the occurrence of DKD at univariate analysis, although BMI was the only significant predictor at stepwise multivariate regression analysis [206].

Gender differences were also noted in the impact of serum uric acid on DKD risk [223,224,225]. In a large Chinese cohort [226], hyperuricemia was independently associated with an increased risk of DKD in both genders, but, after adjustment for traditional DKD risk factors, the association remained significant only in men.

Data on potential gender differences in the response to drugs recommended for DKD are even more sparse. Thus, recent guidelines recommend the use of inhibitors of sodium-glucose cotransporter 1 (SGLT2i) and glucagonlike protein 1 receptor agonists (GLP1Ras) in T2DM subjects with DKD, on the wave of the encouraging results of many cardiovascular outcome trials (CVOTs) and dedicated investigations in patients with CKD with or without T2DM, but very few of them have specifically evaluated sex or gender differences [227,228].

When renal outcomes were investigated as a safety issue in a retrospective cohort study [229], no differences between T2DM male and females in acute kidney injury (AKI) were reported for SGLT2i (female: 20.9 cases/1000 patients; male: 20 cases/1000 patients) and for GLP1Ras (female: 17.8 cases on 1000 GLP-1RA users; male: 36 cases/1000 patients). Furthermore, no sex interaction was reported in another study exploring the risk of serious renal events among SGLT2i- and GLP1Ras- users [230]. Overall, trials with empagliflozin, canagliflozin, dapagliflozin in patients with and without T2DM, also showed no significant sex gender interactions on renal outcomes, indicating a similarly beneficial effect of SGLT2i in the two genders, although the number of women included in the trial population was different among these studies [231,232,233].

Collectively these data indicate that the emerging evidence of a gender-difference in the impact of DKD risk factors is yet to be confirmed, but the literature data consistently indicate that DM women, especially those with T2DM, do not reach targets for major CVD/DKD risk factors as easily as men. The reasons behind these gender differences are still only partly explored, and they may include differences in drug prescriptions, adherence and/or drug response, research areas that should be further explored. Whatever the reason, out-of-target risk factors may contribute to the worst outcomes observed in DM women even in terms of DKD-related mortality [234].

## 6. Conclusions

DKD is one of the most burdensome complications of both T1DM and T2DM. While the prevalence of CVD and other chronic DM complications have shown a progressive decrease in industrialized countries, the prevalence of DKD has not, in spite of the recent diagnostic and therapeutic advances, mostly because of the impact of population ageing and the continuous increase in the prevalence of T1DM and T2DM worldwide. In order to cope with this challenge, the guidelines recommend a personalized approach, where sex and gender differences need to be taken into account.

Sex and gender differences have been reported in the prevalence of specific DKD phenotypes, as well as in the prevalence, impact and control of common DKD risk factors.

It has become well recognized that both albuminuria and eGFR are important factors for DKD diagnosis, with the non-albuminuric phenotype more prevalent in DM women and the albuminuric one in DM men. As for progression toward ESDR, the data indicate a faster progression and overall worse outcomes in DM women, especially those with T2DM in the late decades of life.

Hormonal and genetic factors have also been shown to play a relevant role in explaining these differences in DKD, as demonstrated by several experimental studies, showing an overall protective role for estrogens and progesterone (Figure 1). Conversely, although many genetic loci have been associated with an higher DKD risk both in T1DM and T2DM, their impact seems to vary according to selected gene variants, type of DM, DKD phenotype, study design and according to sex (Table 3). In spite of this increasing evidence, many areas of knowledge still need to be covered. No gender-specific guidance on important diagnostic and therapeutic aspects is available to date. Particularly, it is still not known whether the reported gender differences in DKD phenotypes will result in different outcomes in terms of renal progression and/or CVD mortality. Moreover, the potential protective effect of estrogen replacement therapy on renal outcomes is still under debate. Additionally, the relative impact of socio-economical differences, i.e., the gender disparities, on DKD incidence and progression has not been evaluated so far. Potential sex/gender differences in the therapeutic approach to DKD is another area that needs to be covered. Thus, it is well recognized that women usually experience more drug side effects, and that female gender is poorly represented in randomized controlled trials testing new drugs, an issue that also applies to drugs recommended for patients with DKD, which witness the predominance of the male gender among trials’ participants.

Although the available evidence suggests similar renal benefits and/or side effects from the new hypoglycemic drugs with renal benefit in T2DM men and women, the lower representation of women in these trials and the overall lack of a gender-specific analysis prevent us from drawing firm conclusions on these important efficacy and safety issues.

In conclusion, sex and gender differences embrace several aspects of DKD pathogenesis, diagnosis, and management, leaving a number of unanswered questions that should be addressed by future research in order to reduce the burden of this serious complication.

## Figures and Tables

**Table 1:** Sex/gender differences in DKD phenotypes.

Reference	Ethnic Group	Study Design	Sex Specific Association	MAU	Low eGFR	ESRD
**Studies on T2DM subjects**						
Gall, 1997 [20]	Denmark	Prospective	Male	Higher risk	NR	NR
Lewis J, 2001 [21]	Multi-ethnic	Intervention study	Female	Higher risk	NR	NR
Keane WF, 2003 [22]	Multi-ethnic	Intervention study	Female	Higher risk	Higher risk	Higher risk
Rossing K, 2004 [23]	Denmark	Prospective	Both sexes	NR	Higher risk	Higher risk
Retkaran, 2006 [24]	UK	Prospective	Male/Female	Higher risk male	Higher risk female	NR
Penno, 2011 [25]	Italy	Cross-sectional	Male/Female	Higher risk male	Higher risk female	NR
Yu M, 2012 [26]	USA	Cross-sectional	Male/Female	Higher risk male	Higher risk male	Higher risk female
Jardine, 2012 [27]	UK	Intervention study	Male	NR	NR	Higher risk
Zoppini, 2012 [28]	Italy	Prospective	Both sexes	NR	Higher risk	NR
Altemtan,2012 [29]	UK	Retrospective	Both sexes	NR	Higher risk	NR
Elley, 2013 [30]	New Zealand	Nationwide cohort	Female	NR	NR	Higher risk
de Hautecloque 2014 [31]	France	Prospective	Male	NR	Higher risk	Higher risk
Kaiwara 2016 [32]	Japan	Prospective	Female	NR	Higher risk	NR
**Studies on T1DM subjects**						
Orchard, 1990 [33]	US	Prospective	Male	Higher risk	NR	NR
Lovshin, 1990 [34]	US	Cross-sectional	Male	Higher risk	NR	NR
Holl, 1999 [35]	Germany	Retrospective	Female	Higher risk	NR	NR
Jacobsen, 1999 [36]	Denmark	Prospective	Male	NR	Higher risk	NR
Rossing, 2002 [37]	Denmark	Prospective	Both sexes	Higher risk	NR	NR
Zhang, 2003 [38]	US	Prospective	Male	Higher risk	NR	NR
Hovind, 2004 [39]	Denmark	Prospective	Male	Higher risk	NR	NR
Finne, 2005 [40]	Finland	Register	Male	NR	NR	Higher risk
Sibley, 2006 [41]	US	Prospective	Male	Higher risk	NR	NR
RAile, 2007 [42]	Germany	Prospective	Male	Higher risk	NR	NR
Monti, 2007 [43]	US	Cross-sectional	Both sexes	Higher risk	Higher risk	NR
Mollsten, 2010 [44]	Sweden	Population	Male	NR	NR	Higher risk
Costacou, 2011 [45]	US	Prospective	Male 1950-1964	Higher risk	NR	Higher risk
Costacou, 2011 [45]	US	Prospective	Female1965-1980	Higher risk	NR	Higher risk
Harjutsalo, 2011 [46]	Finland	Prospective	Male	NR	NR	Higher risk
Kautzy-Willer 2013 [47]	Austria	Cross-sectional	both sexes	Higher risk	Higher risk	NR
Skupien, 2019 [48]	Multi-ethnic	Prospective	Male	NR	NR	Higher risk
**Studies including T1DM/T2DM subjects**						
Dick, 1994 [49]	Canada	Prospective	Female	NR	NR	Higher risk
Xue, 2007 [51]	USA	Prospective	Female	NR	NR	Higher risk
Yamagotha, 2007 [52]	Japan	Prospective	Male	Higher risk	Both sexes	NR
Hippsley-Cox,2010 [53]	UK	Registrative data	Female	NR	Both sexes	Higher risk
Hoffman F, 2011 [54]	Germany	Claims data	Female	NR	NR	Higher risk
Johnson, 2011 [55]	USA	Retrospective	Male	NR	NR	Higher risk
Tohidi, 2012 [56]	Iran	Prospective	Female	NR	Higher risk	NR
Kei, 2013 [57]	Japan	Prospective	Both sexes	Higher risk	NR	NR
van Blijderveen, 2014 [58]	Netherlands	Retrospective	Male/Female	Higher risk Male	Both sexes	Higher risk Female
Haroun, 2003 [50]	USA	Prospective	Female	NR	NR	Higher risk F
Ricardo,2018 [13]	USA	Prospective	Male	Higher risk Male	Both sexes	Higher risk Male

Abbreviations: DKD, diabetic kidney disease; eGFR, estimated glomerular filtration rate; MAU, micro/macroalbuminuria; ESRD, End stage renal disease; T1DM, type 1 diabetes mellitus; T2DM, type 2 diabetes mellitus; NR, sex/gender differences not reported.

**Table 2:** Age- and gender differences in DKD prevalence, incidence and progression in T1DM and T2DM participants in AMD Annals Initiative.

**T1DM**			
	**Prevalence**	**Incidence 5 years**	**4 years Progression** eGFR<60 mL/min or >30% reduction
**Male Sex**	DKD 1.01 (0.91–1.11) *p* = 0.901 Low eGFR 0.64 (0.55*–*0.74) *p* < 0.001 MAU 1.26 (1.14*–*1.40) *p* < 0.001	DKD 1.01 (0.81*–*1.27) *p* = 0.913 Low eGFR 0.96 (0.59*–*1.56) *p* = 0.873 MAU 1.04 (0.82*–*1.32) *p* = 0.760	0.59 (0.46*–*0.76) *p* < 0.001
**Age**	**By 10 year** DKD 1.15 (1.10*–*1.19) *p* < 0.001 Low eGFR 1.85 (1.74*–*1.96) *p* < 0.001 MAU 0.93 (0.89*–*0.87) *p* < 0.001	**By 10 year** DKD 1.07 (0.96*–*1.18) *p* = 0.203 Low eGFR 1.95 (1.57*–*2.43) *p* < 0.001 MAU 0.93 (0.84*–*1.04) *p* = 0.227	**By 10 year** 1.46 (1.30*–*1.63) *p* < 0.001
**T2DM**			
	**Prevalence**	**Incidence 5 years**	**4 years Progression** eGFR <60 mL/min or >30% reduction
**Male Sex**	Low eGFR 0.69 (0.64*–*0.73) MAU 1.89 (1.81*–*1.98) Both 1.52 (1.42*–*1.63)	Low eGFR 1.373 (1.326*–*1.422) *p* < 0.001 MAU 1.075 (1.033*–*1.118) *p* < 0.001 Both 1.381 (1.306*–*1.460) *p* < 0.001	**In subjects with DM and hypertension** 0.78 (0.72*–*0.86) *p* < 0.001
**Age**	**By 1 year** Low eGFR 1.12 (1.11*–*1.12) MAU 1.01 (1.01*–*1.01) Both 1.12 (1.12*–*1.13)	**By 10 year** Low eGFR 0.767 (0.681*–*0.864) *p* < 0.001 MAU 1.355 (1.220*–*1.504) *p* < 0.001 Both 1.090 (0.926*–*1.283) *p* = 0.30	**By 10 year** 1.49 (1.41*–*1.58) *p* < 0.001

Abbreviations: DKD, diabetic kidney disease; eGFR, estimated glomerular filtration rate; MAU, microalbuminuria.

**Table 3:** Studies reporting sex specific associations of selected gene variants with DKD phenotypes.

Reference	Ethnic Group	Locus	Study Design	Sex Specific Association *	MAU	Low eGFR	DKD
Studies on T2DM subjects							
Lin, 2009 [191]	US	AGT1R (1166)	cohort study	Male/Female	Not significant	Higher risk	Higher risk
Tien, 2009 [198]	Taiwan	ACE D/I	Prospective	Female	Higher risk	Higher risk	Higher risk
Mooyaart, 2011 [181]	Multi-ethnic	24 gene variants: ACE, AKR1B1 (two variants), APOC1, APOE, EPO,NOS3 (two variants), HSPG2, VEGFA, FRMD3 (two variants), CARS (two variants), UNC13B, CPVL and CHN2, and GREM1, plus 3 variants not near genes.	GWAS	NR	Higher risk	Higher risk	Higher risk
Ahluwalia, 2011 [193]	Sweden	CNDP1 (rs2346061)	Case–control	Male/Female	Higher risk	Not significant	Higher risk
Ahluwalia, 2011 [193]	Sweden	CNDP2 (rs7577)	Case–control	Female	Higher risk	Not significant	Higher risk
Kurashige, 2013 [195]	Japan	CNDP1 (rs12604675)	Case–control	Female	Higher risk	Not significant	Higher risk
Alkhalaf, 2015 [194]	Netherlands	CNDP1 (5L-5L)	prospective	Male/Female	Not significant	Not significant	Not significant
Teumer, 2015 [183]	European	*RAB38*/*CTSC* (rs649529), *HS6ST1* (rs13427836), *CUBN* (rs10795433)	GWAS	NR	NR	NR	NR
Prudente, 2017 [180]	Italy	UMOD (rs12917707)	Cross-sectional	NR	NR	NR	NR
Russo, 2017 [206]	Italy	CETP Taq1B	cohort	Female	Not significant	Not significant	Not significant
van Zuydam, 2018 [184]	European	GABRR1 (rs9942471)	GWAS	NR	Higher risk	Not significant	Higher risk
Huang, 2019 [207]	Taiwan	CETP rs1800775	Cross-sectional	NR	Higher risk	Higher risk	Higher risk
Mantovani, 2019 [205]	Italy	PNPLA3 rs738409	Cross-sectional	Female	NR	Higher risk	Higher risk
Vujkovic, 2020 [189]	Multi-ethnic	UMOD	GWAS	Not significant	Not significant	Higher risk	Higher risk
Studies on T1DM subjects							
Freire, 1998 [190]	US	AGT (M235T)	case–control	Male	Higher risk	Not significant	Higher risk
Miynarski, 2005 [202]	US	CCR5 (A59029G)	Case–control	Male	Higher risk	Higher risk	Higher risk
Miynarski, 2005 [202]	US	CCR5 (32bp deletion)	Case–control	Male	Higher risk	Higher risk	Higher risk
Mollsten, 2011 [192]	Denmark, Finland, France and Sweden	AGTR1 (rs5186)	case–control	Male	Higher risk	Not significant	Higher risk
Gu/Horova, 2012 [204]	European	IGF2 (rs10770125)	Case–control	Male	Higher risk	Higher risk	Higher risk
Gu/Horova, 2012 [204]	European	IGF2BP2(rs4402960)	Case–control	Male	Higher risk	Higher risk	Higher risk
Montero, 2013 [201]	Brazil/France/Belgium	CYBB (rs6610650)	Cross-sectional	Female	Higher risk	Higher risk	Higher risk
Montero, 2013 [201]	Brazil/France/Belgium	GPX4 (rs713041)	Cross -sectional	Male	Higher risk	Higher risk	Higher risk
Saldholm, 2013 [203]	Finland	Chr2q31.1 (rs4972593)	GWAS	Female	NR	Higher risk	Higher risk
Sanholm, 2017 [182]	European	AFF3, CNTNAP2, NRG3, and PTPN13, ELMO1, 13q, and SIK1	GWAS	NR	Higher risk	Higher risk	Higher risk
Gu, 2019 [200]	Sweden	SOX2 (rs11915160)	Case–control	Female	Higher risk	Higher risk	Higher risk
Salem, 2019 [185]	European	COL4A3 (rs55703767)	GWAS	Male	Higher risk	Higher risk	Higher risk
Mori, 2020 [186]	Brazil	HSD11B1 (rs17389016)	cohort	NR	Not significant	Higher risk	Higher risk

Abbreviations: DKD, diabetic kidney disease; * Sex specific association: sex with the strongest association is reported; eGFR, estimated glomerular filtration rate; MAU, micro/macroalbuminuria; T1DM, type 1 diabetes mellitus; T2DM, type 2 diabetes mellitus; NR, sex/gender differences not reported. Not significant, no significant association.
